# Protein aggregate or agglomerate: similar punctate structure with distinct biological profiles

**DOI:** 10.1038/s44320-025-00143-z

**Published:** 2025-09-15

**Authors:** Rui Sun, Yu Liu

**Affiliations:** 1 State Key Laboratory of Medical Proteomics, National Chromatographic R. & A. Center, Dalian Institute of Chemical Physics, Chinese Academy of Sciences, Dalian, 116023 China; 2 University of Chinese Academy of Sciences, Beijing, 100049 China

**Keywords:** Translation & Protein Quality

## Abstract

R. Sun & Y. Liu discuss the study by Levin et al, in this issue of *Mol Syst Biol*, that shows that protein agglomerates are functionally different than protein aggregates and have no major consequences on cellular growth and physiology.

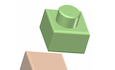

## Teaser

Under various physiological or pathological conditions, biomacromolecules may undergo assembly or misassembly to form puncta-shaped micro-structures in the cell. These assembled bodies contain a cluster of phase-separated proteins or nucleic acids. Therefore, it has long been recognized that disordered, misfolded or aggregated proteins entangled with their cellular interactome dominate in these “dot-like” compartments, exemplified by aggresome, stress granule, p-body, etc. Recently, the field has embarked on understanding their complex biological profiles, including the micro-organization (Ye et al, 2023), composition (Feng et al, 2023), and pathophysiology (Wang et al, 2021). In this work, (Levin et al, 2025) focused on the biological profiles of another type of protein assembly, namely “agglomerate” (Garcia-Seisdedos et al, 2019), induced by mutation of folded-state protein filamentation in yeast cells. In-depth proteomics profiling of cells containing protein agglomerates or aggregates revealed distinct quality control mechanisms in terms of their interactome and proteome-wide perturbations. This work, from a systems biology perspective, highlights the distinct biological profiles (interactome, cell fitness, proteome impact) determined by the protein foldedness inside these phenotypically similar cellular “dots”.

The protein homeostasis (proteostasis) network (Balch et al, 2008), consisting of a collection of molecular chaperones, folding enzymes, protein degradation machineries, is known to rescue or degrade misfolded and aggregated proteins. The authors began their study by asking a scientific question: What are the cellular mechanisms to cope with aberrant, mutation-induced agglomerates and their cellular impacts, compared to the well-known proteostasis surveillance for misfolded aggregates? To this end, they used a collection of previously established mutation-triggered protein assemblies with signature cellular punctate structures. To distinguish the folding states between misfolded aggregation vs folded agglomeration, they employed transmission electron microscopy (TEM) coupled with correlative light electron microscopy (CLEM) on fixed cell sections to clearly show the small bundle-like filaments, indicating their folded nature instead of amorphous aggregates in vivo.

**Figure 1:**
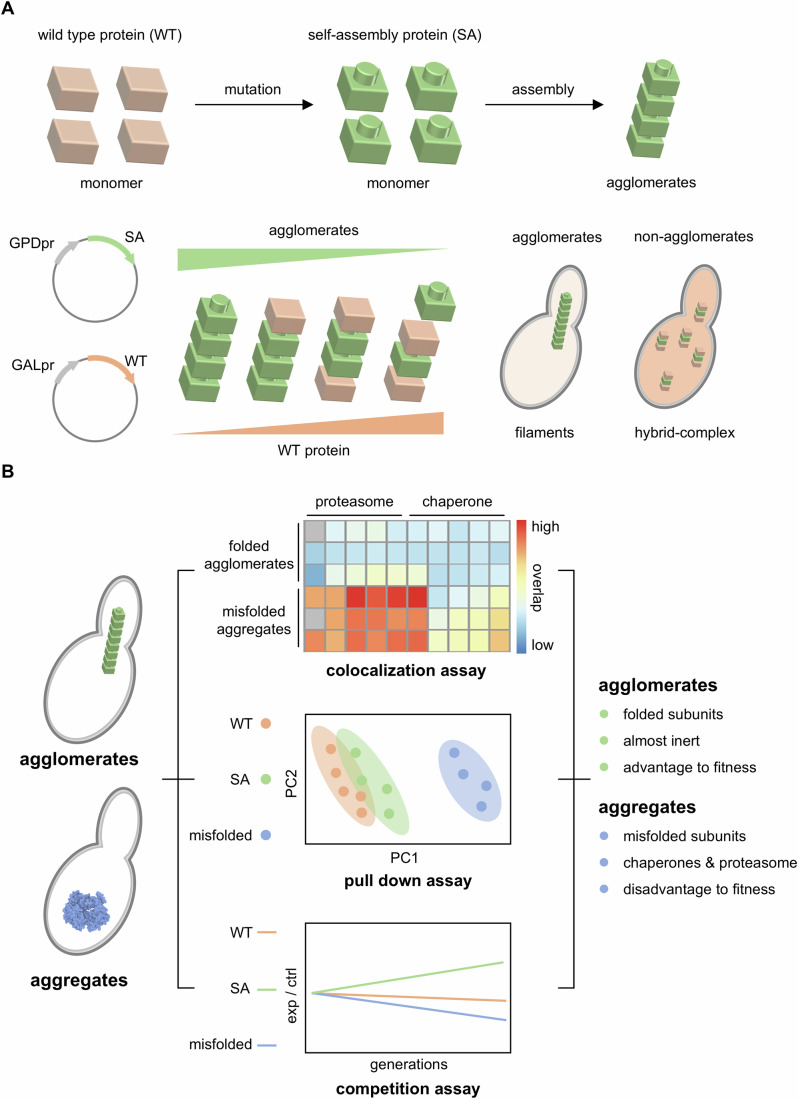
Profiling the biological roles of protein agglomerates in yeast cells. (**A**) Creating mutant-induced self-assembly proteins as an agglomerate model. The mutant proteins assemble to form filaments, whereas the wildtype do not. The mutant protein will co-assemble with the wildtype only if it retains the folded structure. (**B**) Biological profiles of agglomerates versus aggregates. Using colocalization assay, pull-down assay and competition assay, agglomerates were shown to be significantly different from misfolded aggregates. The agglomerates were inert in the cell with negligible interactions with chaperones and the proteasome. Besides, competition growth assay revealed that agglomerates were slightly advantageous to cell fitness but not aggregates.

However, it has always been technically challenging to profile the folding states of a protein-of-interest in live cells (Liu et al, 2014; Liu et al, 2018). In this work, (Levin et al, 2025) devised a facile in vivo imaging assay to test the foldedness of these mutants in live cells, i.e., to distinguish whether they are aggregates or agglomerates. This assay works in the way that expression of a wild-type subunit (fused with mCherry) could co-assemble with the mutant one (fused with YFP) if it retained its folded structure, rescuing them from forming brightly fluorescent puncta or filaments (Fig. 1A). The tendency to form agglomerates was quantitatively reflected by the fluorescence intensity ratio of maximal YFP signal over median averaged YFP across the cell with background normalization. Such a rapid and simple imaging assay allowed them to distinguish agglomeration, aggregation and even the intermediate state in between with strict statistical controls.

Despite similar punctate morphology, it is unclear whether proteostasis network components interact and cope with agglomerates as analogous to aggregates. To this end, the authors colocalized these self-assembling mutants with (1) proteostasis components; (2) autophagy-related proteins; (3) stress-responsive proteins; (4) filamentous assemblies. This constructed imaging library with misfolded protein controls revealed that agglomerates clustered together, but they did not colocalize with proteasome subunits, proteases, or chaperones. In contrast, the misfolded as well as partially misfolded mutants colocalized with proteostasis players, including proteasome components and several cytosolic chaperones.

The finding of mislocalization of agglomerates and proteostasis network components is intriguing. While the interactome of phase-separated proteins has been widely characterized (Pan et al, 2025), the agglomerates’ interacting network remains largely unknown. To systematically assess cellular coping mechanisms for agglomerates, shotgun proteomics was applied to the pull-down interactome samples for wild-type, misfolded, and agglomerated mutants. Principal component analysis (PCA) echoed previous findings, whereby interacting patterns were retained for wild-type, agglomerating, or YFP-only controls but significantly differed from the misfolded variant. A single over-represented protein, cytosolic L-asparaginase that forms cell-spanning filaments, was discovered herein to interact with the agglomerating mutant. Gene Ontology analysis also showed no category as over-represented for agglomerates, but “protein-folding” and “proteolysis” functions for misfolded aggregates. These results from a proteomic perspective profiled the distinct coping mechanisms between agglomerates and aggregates for the cell.

Finally, the authors touched upon the issue of the physiological impact of these agglomerates by testing cell fitness upon expression of the created enzymatically inactive mutants (Fig. 1B). To amplify sensitivity in measuring fitness, (Levin et al, 2025) adopted serial and continuous competition assays to show that the impact of expressing an agglomerating mutant outperformed wildtype and misfolded variant. Such observation leads to another question: how does agglomeration alter or remodel the cellular proteome? Again, shotgun proteomics was used to profile the proteomic landscape of yeast cells upon expressing the wild-type, agglomerating, or misfolded variants for three different proteins. Echoing the above discoveries, proteomics results also showed upregulation of “chaperone-related”, “protein-folding” and “response-to-heat” proteins upon expressing misfolded mutants, indicating active involvement of the proteostasis network for quality control of aggregates. However, the cellular coping mechanism for agglomerates is different but not clear-cut. Upregulation of “cell-wall” and “bud-neck” proteins and downregulation of “metabolic” and “cell polarity and actin polarization” proteins were identified. Given these results, one may speculate that agglomeration-induced large filament structure may disrupt cell organization, sometimes impeding cell division.

As the authors outlooked, the fact that most agglomerates being benign and cell-compatible may open up new avenues for their in-cell synthetic biology applications. These applications span from expression reporters to molecular sensors and protein organization scaffolds.
